# The Impact of the Interventions for 4^+^ Antenatal Care Service Utilization in the Democratic Republic of Congo: A Decision Tree Analysis

**DOI:** 10.5334/aogh.2537

**Published:** 2019-12-27

**Authors:** Hocheol Lee, Sung Jong Park, Grace O. Ndombi, Eun Woo Nam

**Affiliations:** 1 Department of Health Administration, Yonsei University Graduate School, Wonju, KR; 2 Yonsei Global Health Center, Yonsei University, KR; 3 Department of Applied Statistic, Yonsei University Graduate School, Wonju, KR

## Abstract

**Background:**

In 2015, the United Nations set the sustainable development goals (SDGs) with a focus on the maternal mortality ratio (MMR), to decrease the mortality rate of newborns to 70 per 100,000 by 2030. Despite efforts to achieve the SDGs, the MMR in the Democratic Republic of Congo (DRC) was 693 per 100,000 in 2015—the sixth highest in the world and higher than the average (547 per 100,000) of sub-Saharan Africa.

**Objectives:**

The primary aim was to identify effect factors of 4^+^ antenatal care (ANC) of the maternal and child health care (MCH) project focused on reproductive women in the DRC.

**Methods:**

This study used a before and after study design and focused on ANC utilization of reproductive age women in Kenge, DRC. This study provided the MCH intervention based on three phases of the Three Delays Model from 2014 to 2017. We interviewed 2,663 participants from 2014 to 2017. This study used the decision tree node for prediction of 4^+^ ANC utilization.

**Findings:**

The decision tree showed that hand-washing (1.000) was the most important factor for receiving 4^+^ ANC services in the midline I survey, followed by writing skills (0.891), satisfaction with health facilities (0.869), age (0.782), and awareness of interventions (0.621). The results of the midline II decision tree demonstrated that MCH promotion by signboard (1.000) was the most important factor for 4^+^ ANC services, followed by income (0.970), and abortion (0.894). In the third year, distance (1.000) was the most important factor, followed by abortion (0.940) and knowledge of exclusive breastfeeding (0.806).

**Conclusions:**

The most important factors were related to awareness. We recommend conducting interventions focused on improving awareness increase 4^+^ ANC utilization. Sustainability intervention for improving the 4^+^ ANC utilization requires that focus on the infrastructure, such as accessibility and knowledge, of reproductive women.

## I. Introduction

The maternal mortality ratio (MMR), the number of maternal deaths per 100,000 live births, has been reduced by an average of 2.3% per year from 1990 to 2015. Despite millennium development goals (MDG) efforts, 830 women die every day from complications related to pregnancy or childbirth around the world. Almost 99% of MMR occurs in developing countries, and half of all MMR occur in sub-Saharan Africa [1,2,3]. The MMR was 693 in the Democratic Republic of Congo (DRC) in 2005, which was the sixth lowest developing country [4,5,6].

Previous studies have shown that antenatal care (ANC) services are the most cost-effective intervention for reducing the MMR in developing countries [7]. WHO defined ANC as the care provided by skilled health care experts to pregnant women in order to ensure the best health conditions for both mother and baby during pregnancy [8]. WHO and a previous study suggested that essential ANC interventions should be provided more than four times (4^+^) [9,10]. Additionally, ANC services can provide guidance on other health risk factors, such as non-communicable diseases (NCD), smoking, alcohol consumption, obesity, and malnutrition [11,12]. According to a meta-analysis of 40 articles, which examined studies centered in Ethiopia, women who had experienced ANC services were more likely to receive ANC services as well as postnatal care (PNC) services [13]. ANC services have effects not only on mothers but also on their children. Further, ANC service is an important factor associated with parenting skills [14]. According to Poma’s study, if more women get ANC services, the MMR could decrease 28% [15].

Globally, 71% of women receive ANC services and 95% of women in developed countries have access to ANC [3]. On the other hand, only 69% of women have at least one ANC visit in sub-Saharan Africa, and only 44% of them have 4^+^ ANC services [3].

The Ministry of Public Health (MoPH) in the DRC recommended that pregnant women receive 4^+^ ANC services continuously, and MoPH also support ANC policies [16]. Despite MoPH support, DRC their Mother and Child Health (MCH) indicator has decreased, which includes ANC service utilization, MMR, PNC service utilization, and others, of reproductive women owing to civil war [17]. WHO recommended interventions for improving the health status of reproductive women, which was health education, providing general health information, transportation system for referrals, etc. [18].

The aims of this study were to identify the effect factors associated with 4^+^ ANC services of the MCH project with a focus on women of reproductive age in the DRC. Additionally, this study aims to identify the change in the effect factors each year after providing the MCH project intervention.

## II. Materials and Methods

### 1. Study design

This study was conducted using a community-based study from February 2014 to July 2017 for women of reproductive age in DRC. Study design was based on the Three Delays Model [19].

The study was conducted in Kenge city in the Kwango district, which is located about 120 km east of Kinshasa, the capital of the DRC. During the study, about 55,000 people lived in Kenge city. Kenge city has one hospital and 22 primary health centers. One of the health centers has 20 Resilient Communities Organization (RECO) community health workers, who deliver basic public health programs such as reproductive health, hygiene, and family planning education.

### 2. Sample size and data collection

The sample size was calculated using the Raosoft at a 95% confidence interval, 5% error range. The sample was selected through probability proportionate to size in each health areas (Aires de Santé; AS) in Kenge city. We collected data from 30 households per AS based on the Central Limit Theorem. The data were collected during home visits with women of reproductive age through face-to-face interviews conducted by enumerators. During the visit, enumerators informed the participants about the study and obtained informed consent from each respondent. Emphasis was placed on the respondents’ rights to refuse to answer any of the questions during the visit. Each survey team consisted of a team manager, data quality supervisor, and two enumerators. The questionnaire took about one hour per woman. As a result, we interviewed 602 participants in 2014, 622 participants in 2015, 720 participants in 2016, and 719 participants in 2017. Any data with errors were deleted, which left data from 461 participants in 2014, 592 in 2015, 685 in 2016, and 693 in 2017.

### 3. Study instruments

The questionnaire used was a modified version of the UNICEF Multiple Indicator Cluster Surveys 2011(MICS; 2011). This questionnaire was revised three times with three health experts from the School of Public Health/University of Kinshasa (UNIKIN) in the DRC. Thereafter, the questionnaire was validated by conducting a pre-survey among 36 reproductive women in Maluku city, which is similar demographically to Kenge city.

### 4. Intervention

This study provided interventions according to the three phases of the Three Delays Model. In the first phase, educational program intervention was provided to reproductive women to increase awareness and promote MCH services at health facilities [19]. In the second phase, emergency transportation was provided via ambulance to a secondary hospital and by motorcycle to each health centers. In the third phase, this study provided a capacity building program for health workers to improve the quality of health services. This training program cooperated with Programme National la Santé de la Reproduction (PNSR), Programme National de Lutte contre les Infections Respiratoires Aigues (PNIRA), Programme National d’Approvisionnement en Médicaments essentiels (PNAME) and Programme National de Nutrition (PRONANUT), which are public health education organizations in the DRC.

### 5. Variables

The dependent variable was the number of the ANC services that were utilized during pregnancy. To measure it, the questionnaire asked was “How many times did you visit a health facility for ANC before your last delivery?” If respondents had received more than 4 ANC services at a health center then they were coded as “1”, all others below four visits were coded as “0”.

This study selected the independent variable based on the Anderson model including predisposing, enabling, and need factors for 4^+^ ANC services [20]. This study selected the independent variable from among predisposing factors such as age, number of household members, educational level, reading skills, writing skills, knowledge of maternal and child health, monthly household income, and health insurance [20,21,22,23,24,25,26].

Enabling factor variables were selected, including promotion by radio broadcasting, source of MCH awareness (such as a community leader, signboard, radio, etc.), distance to the health facility, RECO activities, type of delivery, and hand-washing practice [23,27,28,29]. This study selected the independent variable from among need factors such as frequency of childbirth, abortion, hand-washing, awareness of the importance of exclusive breastfeeding, ANC, delivery at health facilities, satisfaction with the services at the health facilities, and satisfaction with the health facility setting [7,20,30,31].

### 6. Statistical analysis

The SAS Enterprise Miner Workstation version 14.2 was used to analyze the data. This study used a decision tree node, which is the most representative classification model of data mining, to predict 4^+^ ANC utilization. The decision tree involves classification and prediction by using a node structure. In this study, the decision tree was created by repeatedly dividing the data by each leaf node [32]. As opposed to linear regression, logistic regression, and neural network models, the decision tree is a non-parametric method without common assumptions such as linearity, nominally, and equal variance [33]. In this study, the decision tree model involved cross-validation by dividing the training data (70%) and the validation data (30%). The decision tree algorithm was the chi-squared automatic interaction detection (CHAID) for a split node. CHAID used the chi-square test to determine the best-fit split during the tree-growing process [34]. The model’s goodness of fit test used misclassification and average squared error [35].

### 7. Ethical consideration

All three parts of this survey were annually approved by the Institutional Review Board (IRB) of Yonsei University in Korea and the Kinshasa University Bioethics Review Board (BRB). Each document had IRB numbers—1041849-201406-BM-027-01, 1041849-201406-BM027-02, and 1041849-201406-BM-027-03. Kinshasa University’s BRB numbers were ESP/CE/021/14. ESP/CE/021/2015, and ESP/CE/057/2016. Informed consent was obtained from each respondent before information was collected.

## III. Results

### 1. Characteristics of the respondents

Table 1 shows the ANC services, demographics such as age, monthly income, number of households, writing skills, education level, frequency of pregnant, childbirth from baseline (2014) to endline (2017). Approximately 41.4% of respondents had experienced the more than four times ANC services in baseline (2014), 58.8% in midline I (2015), 41.6% in midline II (2016), 53.4% in endline (2017).

**Table 1:** Characteristics of respondents for 4 years from baseline (2014) to endline (2017).

	Baseline N = 461 (%)	Midline I N = 592 (%)	Midline II N = 685 (%)	Endline N = 693 (%)

ANC Services				
More than 4 times	191 (41.4)	348 (58.8)	285 (41.6)	370 (53.4)
Less than 4 times	270 (58.6)	244 (41.2)	400 (58.4)	323 (46.6)
Respondent Age (Mean ± SD)	29.7 ± 7.2	29.4 ± 6.9	29.2 ± 7.2	29.5 ± 7.0
Monthly Income USD (Mean ± SD)	49.4 ± 83.8	57.2 ± 55.2	45.4 ± 78.1	58.0 ± 75.6
Number of Household				
≤6	424 (70.4)	380 (61.1)	481 (67.8)	442 (61.5)
7–10	161 (26.7)	224 (36.0)	211 (29.8)	257 (35.7)
≥11	17 (29)	18 (2.9)	17 (2.4)	20 (2.8)
Writing Skills				
Yes	376 (62.5)	412 (66.2)	448 (62.7)	442 (61.5)
No	226 (37.5)	210 (33.8)	267 (37.3)	227 (38.5)
Education				
Preschool	N/A	29 (4.8)	44 (6.3)	44 (6.3)
Primary School	N/A	60 (9.9)	50 (7.2)	60 (8.6)
Secondary School	N/A	435 (71.8)	504 (72.5)	498 (71.3)
≥Higher Education	N/A	82 (13.2.5)	97 (14.0)	96 (13.8)
Number of Pregnancies (Mean ± SD)	3.7 ± 2.1	4.4 ± 2.4	3.9 ± 2.2	4.2 ± 2.4
Number of Births (Mean ± SD)	3.5 ± 2.1	4.1 ± 2.3	2.6 ± 2.1	3.9 ± 2.3

### 2. Decision tree analysis

The decision tree model’s goodness of fit was analyzed by the misclassification and average squared error in Table 2 [36]. In midline I, misclassification was 0.348 in train and 0.380 in validation. Misclassification in midline II was 0.388 in train and 0.388 in validation. In endline, misclassification was 0.385 in train and 0.395 in validation.

**Table 2:** Decision tree model’s goodness of fit.

	Midline I (2015)		Midline II (2016)		Endline (2017)	

	Train	Validation	Train	Validation	Train	Validation

Misclassification	0.348	0.380	0.388	0.388	0.385	0.395
Average Squared Error	0.224	0.239	0.231	0.234	0.234	0.242

**Figure 1:**
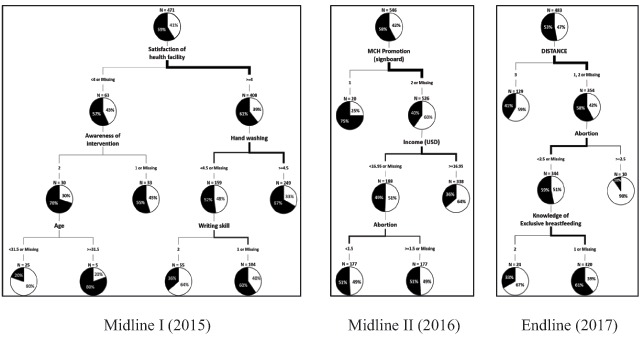
The result of decision tree nodes from midline I (2015) to endline (2017).

In terms of the ranking of variables in the decision tree (Table 3 and Figure 1), hand-washing (1.000) was the most important factor for receiving 4^+^ ANC services in the midline I survey, followed by writing skills (0.891), satisfaction with health facilities (0.869), age (0.782), and awareness of interventions (0.621). The results of the midline II decision tree demonstrated that MCH promotion by signboard (1.000) was the most important factor for women to receive 4^+^ ANC services, followed by income (0.970) and abortion (0.894). In the third year of the MCH intervention, distance (1.000) was the most important factor, followed by abortion (0.940) and knowledge of exclusive breastfeeding (0.806).

**Table 3:** Variable importance rank of decision tree model.

Rank	Midline I (2015)		Midline II (2016)		Endline (2017)	

	Variable	Importance	Variable	Importance	Variable	Importance

1	Hand-washing	1.000	MCH Promotion by Signboard	1.000	Distance	1.000
2	Writing Skills	0.891	Income	0.970	Abortion	0.940
3	Satisfaction with Health Facility	0.869	Abortion	0.894	Knowledge of Exclusive Breastfeeding	0.806
4	Age	0.782				
5	Awareness of Intervention	0.621				

## IV. Discussion

This study is one of the very few studies to identify the effective factors that are associated with 4^+^ ANC services utilization among women of reproductive age in the DRC. In a previous study, 22.9% reproductive women received 4^+^ ANC services in DRC [17]. However, this study shows that up to 41.4% in 2014, 58.8% in 2015, 41.6% in 2016, 53.4% in 2017 had experienced 4^+^ ANC services, which was higher than previous findings (22.9%).

The decision tree approach that was adopted in this study led to the identification of the effective factors on the MCH project from baseline to endline. According to the decision tree analysis, hand-washing was the most important factor in the first year of the intervention. According to the results of a previous study in Uganda, while sanitary education had a significant effect on 4^+^ ANC services among reproductive women, hand-washing did not [29]. We presented health education detailing the infection risks to newborns simultaneously with hand-washing education. This is why we believe hand-washing proved to be the most important factor in the first year of the intervention. According to a study in Sudan, reproductive women with low education levels were less likely to utilize ANC services, and that this was a primary reason for the increase of the MMR [37]. However, the results of this study showed that reproductive women’s writing skills, and not their education levels, were the second most effective factor for 4^+^ ANC service utilization. In a study in Senegal, women who were satisfied with their health facilities were more likely to utilize ANC services [38]. Likewise, the results of this study showed that health facility satisfaction was a significant factor in utilizing 4^+^ ANC services.

In the 2^nd^ year of the intervention, MCH promotion by signboards was the most effective factor for 4^+^ ANC service utilization. In previous studies, low-income families showed low satisfaction with the services of midwives [39]. This study showed that household income was the second most effective factor for women receiving 4^+^ ANC services during the second year of the intervention, which was the midline II survey. Satisfaction with the health facility was the third most effective factor for women receiving 4^+^ ANC services in the first year of the intervention, which was the midline I survey. Even if the satisfaction with health facilities included all health workers, such as doctors, nurses, and midwives, there is a need for future studies to identify whether the satisfaction with the medical services of midwives has an effect on 4^+^ ANC service utilization by reproductive women in the DRC. In addition, abortion was the third most effective factor in the second year of the intervention. According to a study on reproductive health in Cameroon, the frequency of ANC services had an effect on abortion, and the effective factors of abortion were frequent sexual relations and frequency of childbirth [40]. However, the frequency of childbirth had no significant effect on 4^+^ ANC service utilization in the present study.

The decision tree showed that distance from the health facility was the most important factor in the third year of the intervention, which was the endline survey. According to a previous study in Kenya, every 5 km increase in the distance to a health facility made reproductive women 0.25-fold less likely to visit [41]. This study measured within 5 km, 5–10 km, and 10 km distance from the health facilities. As a result, distance proved to be an important factor in the utilization of 4^+^ ANC services in the third year of the intervention. Previous studies have shown that among the various interventions for improving community health – such as agricultural, economic, educational, and infastructure – in the Millennium Villages project from 2010 to 2015, MCH interventions were the most effective, with the typical indicators including ANC, exclusive breastfeeding, and access to safe drinking water [42]. The present study showed that knowledge of exclusive breastfeeding was the third most important factor in the third year of the intervention.

This study had some limitations. First, this study was a before and after study at the community level for four years. However, this survey did not follow the same women each year from 2014 to 2017. In future studies, it is necessary to use a cohort design, which can follow up with the same respondents from baseline to endline. Second, the decision tree model was deemed fitting for data from Africa because it was less affected by outliers and did not require parameter estimation and linearity [36,43]. However, previous studies were conducted comparing models including associations, clustering, neural networks, and logistic regression for classification models. In future studies, it is necessary to conduct services through a comparison of classification models including associations, clustering, and neural networks to identify the effective factor of 4^+^ ANC services in the DRC. Third, this study was conducted by focusing on the cross-sectional effects of the MCH interventions each year. The study took a global perspective on ending poverty and health problems in developing countries. In future studies, it is necessary to focus on national policy strategies and the sustainability of the intervention.

## V. Conclusion

This study aims to identify the effect factors of 4^+^ ANC services of the Korea International Cooperation Agency MCH project focused on women of reproductive age in the DRC for four years. The important factors for 4^+^ ANC services utilization in the first year of the intervention were hand-washing, writing skills, satisfaction with health facilities, age, and awareness of interventions, in that order. The important factors in the second year were MCH promotion by signboards, income, and abortion, respectively. The important factors in the third year were distance, abortion, and knowledge of the importance of exclusive breastfeeding, in that order. As most of the important factors to increasing the number of ANC visits were related to awareness, we recommend conducting an intervention focused on improving women’s awareness to items such as hand-washing, increased program promotion, and general knowledge of MCH. Sustainability intervention for improving the 4^+^ ANC utilization also requires a focus on the infrastructure, such as accessibility and knowledge of reproductive women in DRC, because effect factors in the third year were distance, knowledge, and promotion, which was related to accessibility of geological/information. Therefore, infrastructures should be considered as a focus for sustainability.

## Abbreviations

4^+^ ANC: more than four times antenatal care; AS: Aires de santé; CHAID: Chi-squared Automatic Interaction Detection; KOICA: Korea International Cooperation Agency; MCH; Maternal and child health care; MDG: Millennium Development goals; MoPH: Ministry of Publish Health; MMR: Maternal Mortality Ratio; MNU: Maternal and Newborn care Unit; NCD: Non-communicable disease; PHC: Primary Health Care; PNC: Postnatal care; PNAME: Programme National d’Approvisionnement en Médicaments essentiels; PNIRA: Programme National de Lutte contre les Infections Respiratoires Aigues; PRONANUT: Programme National de Nutrition; PNSR: Programme National la Santé de la Reproduction RECO: Relais Communautaires; SDGs: Sustainable Developments Goals.
